# Can one-step reinforcement learning guide optimal timing for PEG and tracheostomy in severe TBI? Insights from a 2016–2023 retrospective cohort study at a single academic institution

**DOI:** 10.3389/fneur.2025.1700064

**Published:** 2025-11-13

**Authors:** Shrinit Babel, Jade Vanderpool, Maurice Inkel, Milad Behbahaninia

**Affiliations:** 1 University of South Florida College of Medicine, Tampa, FL, United States; 2 Tampa General Hospital, Tampa, FL, United States

**Keywords:** traumatic brain injury, tracheostomy, PEG, reinforcement learning, clinical decision support

## Abstract

**Background:**

Acute management of traumatic brain injury (TBI) presents several challenges in hospital resource planning. While early tracheostomy (trach) and percutaneous endoscopic gastrostomy (PEG) tube placement may improve patient outcomes, the optimal timing and selection criteria for these interventions remain unclear. This study evaluates the impact of PEG and trach timing on key clinical outcomes and applies one-step reinforcement learning (RL) to recommend intervention timing.

**Methods:**

This retrospective cohort study included 263 adult intensive care unit inpatients (194 men, 69 women, age range 18–87), diagnosed with severe TBI requiring trach and/or PEG between 1 January 2016 and 31 December 2023, at a single academic institution. Key outcomes included ICU and hospital length of stay (LOS), complications, time to oral feeding/decannulation, readmission, and mortality. One-step temporal difference (TD) learning and Q-learning were used to predict the expected value of interventions and to recommend optimal timing based on patient states, respectively.

**Results:**

Early PEG and trach interventions were associated with significantly shorter ICU and hospital length of stay (LOS) and fewer complications. Delayed PEG placement, however, was associated with a 67% reduction in the odds of mortality (OR: 0.33, *p* = 0.033) compared to early placement, despite having more total complications. One-step RL suggested greater cumulative rewards with earlier intervention and successfully recommended the optimal day for PEG/trach intervention based on initial patient presentation.

**Conclusion:**

Early interventions are associated with improved outcomes; however, delaying PEG or trach placement may be advantageous in select situations to reduce mortality. RL techniques, such as TD and Q-learning, can aid in decision-making regarding interventions.

## Introduction

Traumatic brain injury (TBI) is a leading cause of mortality and long-term disability globally, with approximately 69 million new cases every year (1). An estimated 10–15% of all TBI cases are classified as moderate to severe based on the Glasgow Coma Scale (GCS) (2). Patients with moderate to severe TBI typically require specialized intensive care management, including mechanical ventilation and nutritional support (2). This can lead to the placement of percutaneous endoscopic gastrostomy (PEG) tubes for enteral feeding or tracheostomies (trach) in cases of prolonged mechanical ventilation. In severe TBI, indicated by a GCS of 8 or less, timely feeding and airway interventions are key issues in improving patient outcomes (3). The timing and contexts of these interventions present substantial challenges and complexities in their acute management (1).

Timely and effective trach placement and nutrition are important in the management, but the optimal timing and assessment of these procedures remain ambiguous. Particularly, there are no standardized guidelines for the optimal timing of trach placement in these patients (4). This is compounded by the lack of consensus and ambiguity in the existing literature, with multiple studies pointing out that trach timing is often left to the physician’s discretion (5).

Some studies have demonstrated that early trach initiation does contribute to better outcomes in terms of the duration of mechanical ventilation and ICU stay. A meta-analysis by de Franca et al. showed that early trach can reduce the duration of mechanical ventilation, ICU LOS, and hospital LOS and lower the risk of ventilator-associated pneumonia (VAP) (6). Similar correlations with length of stay and days of mechanical ventilation have been observed in other studies (7, 8).

Regardless, the association between early trach and overall hospitalization duration, the incidence of pneumonia, and the mortality rate remains inconclusive (9). Others have pointed to a larger risk of complications such as intracranial hypertension or even hospital mortality, with an early trach (10–12). For example, Kocaeli et al. found that early trach placement could increase intracranial pressure and, therefore, complicate patient recovery (11). Contrary to the study by de Franca et al., some retrospective analyses have suggested that trach performed within 24 h was not associated with improved survival but was instead linked to higher rates of pneumonia and longer ICU stays compared with a trach performed within 48 h (13).

TBI patients who undergo trach often require additional support for nutrition and feeding. Percutaneous endoscopic gastrostomy (PEG) has emerged as the preferred method for providing long-term nutrition, especially in patients with prolonged dependence on enteral feeding (14). Indeed, studies have found that early PEG placement within 7 days may reduce hospital length of stay and costs, although there are concerns about worsened outcomes (15, 16). Studies have especially supported simultaneous PEG in neurocritical patients requiring trach, pointing to lower intensive care unit (ICU) and overall hospital stays (17–19). Meanwhile, Chaudhry et al. found that the standard timing for PEG placement (7–14 days) maximized patient outcomes, while both early and late PEG placements otherwise had higher risks of complications, infection, and mortality (20). Delaying PEG placement has also been found to result in fewer complications, especially in select populations (16).

The choice to perform a PEG or a trach involves balancing the potential benefits of early intervention with the risk of an unnecessary procedure. As the literature shows, this decision relies on clinical experience and patient-specific factors; determining the ideal timing for conducting these procedures when warranted adds another layer of complexity. For example, a prospective study by Figueiredo et al. described how PEG placement was often requested too late in half of the reviewed cases. Despite a trend favoring earlier placement, they nevertheless could not decipher a single variable that could reliably predict complications or provide insights into placement. These procedures are key to the long-term recovery of TBI patients, yet their optimal placement is elusive given the complexity and variability of each patient’s condition.

A data-driven approach could provide insights into personalizing the timing of these interventions toward maximizing patient recovery and outcomes. Reinforcement learning (RL) is a branch of machine learning that interacts with a dynamic database to learn optimal actions (21). Specifically, temporal difference (TD) learning and Q-learning—two related RL methods—can be used to estimate the long-term expected rewards of clinical interventions based on historical data and subsequently recommend them to clinicians. TD learning learns from the differences between predicted and actual patient outcomes after an intervention to generate a value function iteratively. Q-learning is an extension of this by not only estimating the expected value of each intervention but also determining the best action in a given patient state.

These techniques have been successfully applied previously in other medical fields, such as oncology, where Zhao et al. used similar techniques to personalize treatment strategies for cancer patients based on historical clinical data and in critical care medicine (22, 23). Similarly, RL can be used as a clinical decision-support system to optimize the timing of critical interventions in the management of TBI patients (23).

Therefore, this study aims to evaluate the impact of the PEG or trach intervention timing on key clinical outcomes in TBI patients. Subsequently, we will apply RL techniques, such as TD learning and Q-learning, to identify personalized, data-driven recommendations for the timing of such interventions that improve outcomes across length of stay, complications, mortality, and readmission.

## Methods

This study involves a retrospective database review of adult patients who suffered TBI and were admitted to Tampa General Hospital (TGH) between 1 January 2016 and 31 December 2023. The objective is to evaluate the outcomes associated with early versus late initiation of enteral feeding and trach tube placement. The specific eligibility criteria are tabulated in Table 1.

**Table 1:** Inclusion and exclusion criteria for the study population.

Inclusion criteria	Exclusion criteria
Patients aged 18 years and older. Admitted to TGH with a diagnosis of severe TBI (admission GCS of 8 or less) due to a blunt mechanism. Underwent trach and/or feeding tube placement (percutaneous or surgical) during their stay.	Patients with a non-surgical feeding tube. Patients who are minors (<18 years old), incarcerated, or pregnant.

### Data collection

Data were collected by extracting and reviewing electronic medical records from TGH’s EpicLink system. The retrospective review included demographic data (age and gender); clinical data such as admission and discharge GCS scores, Injury Severity Score (ISS), neutrophil-to-lymphocyte ratio (NLR), and comorbidities; procedural data such as the type of the procedure (PEG, G-tube, and trach), performing service/team (Acute Care Surgery, Neurosurgery, Interventional Radiology, and ENT), and time from admission to the procedure; and outcomes including ICU and hospital length of stay, time to oral feeding, time to decannulation, complications (pneumonia, bleeding, dislodgement, stress gastric ulcer, and gastric perforation), re-hospitalization rates, short- and long-term post-procedure morbidity and mortality, and disposition at discharge.

### Outcome measures

The data were recorded in a de-identified, aggregate form, with key comparisons being the early (0–7 days), standard (8–14 days), and late (>15 days) placement of the PEG tube, and the early (0–7 days), standard (8–14 days), and late (>15 days) trach. The ICU and hospital length of stay, complications, and time to oral feeding/decannulation are considered for stratification.

### Ethics statement

The patient care team made decisions regarding the placement of the PEG tube, the initiation of oral feeding, the placement of the trach, and the decannulation. This study protocol received approval (IRB ID: STUDY006995 USF IRB) by the Institutional Review Board (IRB) and met the criteria for exemption from IRB review. A waiver of HIPAA authorization was also granted for this retrospective chart review, and a waiver of consent has been approved, given the study’s retrospective nature.

### Statistical analysis

Baseline characteristics for the PEG and trach intervention groups were categorized by intervention timing. Means and standard deviations were used to summarize continuous variables [e.g., age, BMI, GCS, ISS, NLR, and Charlson Comorbidity Index (CCI)], and the Kruskal–Wallis test was used to compare their distributions across the three intervention groups. Categorical variables such as gender were summarized by counts and percentages and subsequently analyzed with the chi-squared test.

The Kruskal–Wallis test was performed to determine whether the distributions of ICU LOS and hospital LOS, total complications, and days to oral intake/decannulation differed significantly across the intervention timing groups; this was followed by post-hoc pairwise comparisons using Dunn’s test with Bonferroni adjustment. Logistic regression models were used to understand the relationship between intervention timing and binary outcomes such as mortality, readmission, and VAP for trach patients. Each model included covariates such as age, gender, ISS, GCS, NLR, and CCI. Odds ratios (ORs) and their 95% confidence intervals (CIs) were reported. Similarly, ordinary least squares (OLS) regression was used for continuous outcomes, such as time to oral intake, time to decannulation, and the change in GCS scores from admission to discharge (delta GCS). Cox proportional hazards were applied to assess time-to-event outcomes, including time to readmission and time to mortality, and were assessed against covariates. Hazard ratios (HRs) and 95% CI were computed. The statistical analysis was conducted on SPSS 29 and Python 3.12.

### Temporal difference learning

A temporal difference (TD) learning algorithm was applied to predict the long-term expected value of interventions based on patient outcomes. This reinforcement learning method updates each intervention group’s value function, $V(S_{t})\;$ , as new outcomes become available. The value function $V(S_{t})\;$ for each group was initialized to zero and iteratively updated by the following:


$$V(S_{t})\leftarrow V(S_{t})+\alpha [R_{t+1}+\mathrm{\gamma V}(S_{t+1})-V(S_{t})],$$

where $S_{t}$ is the current intervention group (early, standard, delayed), α=0.1 is the learning rate, $R_{t+1}$ is the computed reward based on patient outcomes, γ=0.9 is the discount factor, and $V(S_{t+1})$ is the estimated value of the upcoming state within the same intervention group. The reward function *R* was designed to reflect the clinical desirability of select outcomes, such as ICU LOS and hospital LOS, readmission, mortality, VAP (in trach patients), and days to oral intake/decannulation. Reward magnitudes were set heuristically and were expert-defined. To assess the robustness of the TD outputs from these expert-defined weights, we performed two sensitivity analyses and recomputed the TD value functions for each setting. First, we assessed the impact of uniformly scaling all reward magnitudes; second, we varied a single reward component across plausible bounds while holding others fixed. For each configuration, we computed the value-function gap.

A Q-learner was subsequently adapted from the value function to discover the optimal timing for PEG and trach interventions. The state space consisted of patient and demographic variables: age, gender, ethnicity, BMI, ISS, GCS, NLR, and CCI. The action space consisted of possible days for the PEG/trach procedure, from 0 to 30 days after admission. The Q-learner, such as value functions, aims to learn the optimal timing for an intervention by updating Q-values for each state-action pair:


$$Q(S_{t},A_{t})\leftarrow Q(S_{t},A_{t})+\alpha (R_{t+1}+\gamma \max_{a}Q(S_{t+1},a)-Q(S_{t},A_{t})),$$

where $Q(S_{t},A_{t})$ represents the Q-value for forming action $A_{t}$ (i.e., the day of the procedure) in state $S_{t}$ (i.e., patient characteristics defined by state space), $R_{t+1}$ is the computed reward based on patient outcomes, and $\max_{a}Q(S_{t+1},a)$ is the maximum Q-value for the upcoming state, representing the best possible future action. The Q-learning algorithm used an epsilon-greedy policy to balance the exploration of potential intervention timings with the selection of the best-known timing. The exploration rate ϵ decayed over time to favor more exploitation as the model gained confidence in determining an optimal intervention timing.

Bootstrapping was used to compute the confidence intervals for the optimal Q-values for PEG and trach patients. The rigor of the Q-learner was visualized by plotting the average reward over time for both PEG and trach interventions. The RL algorithms were deployed using Python 3.12 and standard packages available on Anaconda.

## Results

A total of 263 patients qualified for the retrospective analysis, aged 18 to 87, with 194 men and 69 women. PEG was performed in 232 patients (Table 2), and trach was conducted in 214 patients (Table 3).

**Table 2:** Baseline characteristics of patients undergoing PEG across early, standard, and delayed intervention groups.

Variable	Early Mean (SD) (*n* = 59)	Standard Mean (SD) (*n* = 79)	Delayed Mean (SD) (*n* = 94)	*p*-value
Age	41.27 (16.48)	43.33 (19.52)	47.18 (20.60)	0.253
Male, *n* (%)	46 (78.0%)	60 (76.0%)	67 (71.28%)	0.614^†^
BMI	27.25 (9.12)	24.18 (9.30)	24.43 (6.88)	0.149
GCS	6.14 (1.52)	6.38 (1.62)	6.45 (1.71)	0.904
NLR	3.11 (2.45)	3.29 (2.31)	3.17 (2.63)	0.609
Charlson Comorbidity Index	1.46 (0.88)	1.60 (0.97)	1.87 (1.02)	0.091
ISS	31.25 (13.12)	31.43 (10.48)	25.94 (11.35)	0.003*
Trach performed, *n* (%)	49 (83.1%)	70 (88.6%)	64 (68.1%)	0.003^†^

Values are presented as mean and standard deviation (SD) unless otherwise specified. * denotes significance. ^†^denotes the chi-squared test for categorical variables.

**Table 3:** Baseline characteristics of patients undergoing trach across early, standard, and delayed intervention groups.

Variable	Early Mean (SD) (*n* = 76)	Standard Mean (SD) (*n* = 79)	Delayed Mean (SD) (*n* = 59)	*p*-value
Age	38.81 (16.42)	41.91 (17.66)	41.76 (19.24)	0.614
Male, *n* (%)	55 (72.37%)	66 (83.54%)	43 (72.88%)	0.188^†^
BMI	26.58 (9.05)	25.00 (7.47)	25.64 (9.66)	0.688
GCS	5.96 (1.58)	5.53 (1.57)	6.31 (1.71)	0.413
NLR	3.09 (2.30)	3.28 (2.27)	3.20 (2.57)	0.654
Charlson Comorbidity Index	1.36 (0.81)	1.58 (0.89)	1.67 (0.94)	0.569
ISS	31.61 (11.91)	31.77 (10.45)	28.76 (9.35)	0.309
PEG performed, *n* (%)	63 (82.9%)	69 (87.3%)	51 (86.4%)	0.71^†^

Values are presented as mean and standard deviation (SD) unless otherwise specified. * denotes significance. ^†^ denotes the chi-squared test for categorical variables.

**Figure 1:**
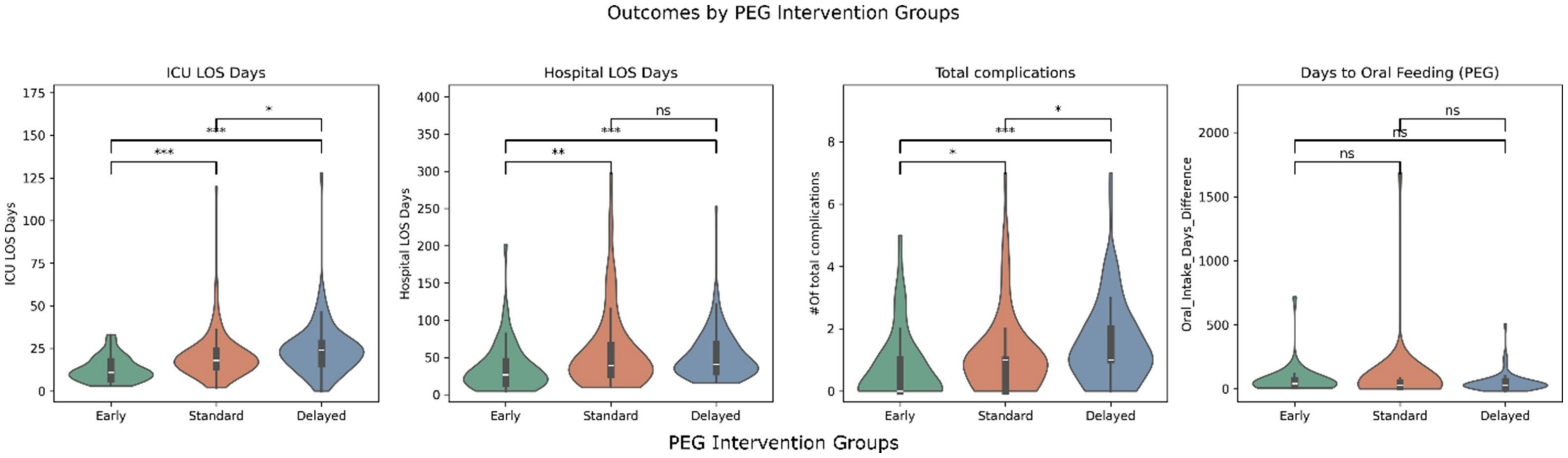
Outcomes by PEG intervention groups (early, standard, and delayed). *** = *p* < 0.001, ** = *p* < 0.01, *p* < 0.05, ns, not significant.

**Figure 2:**
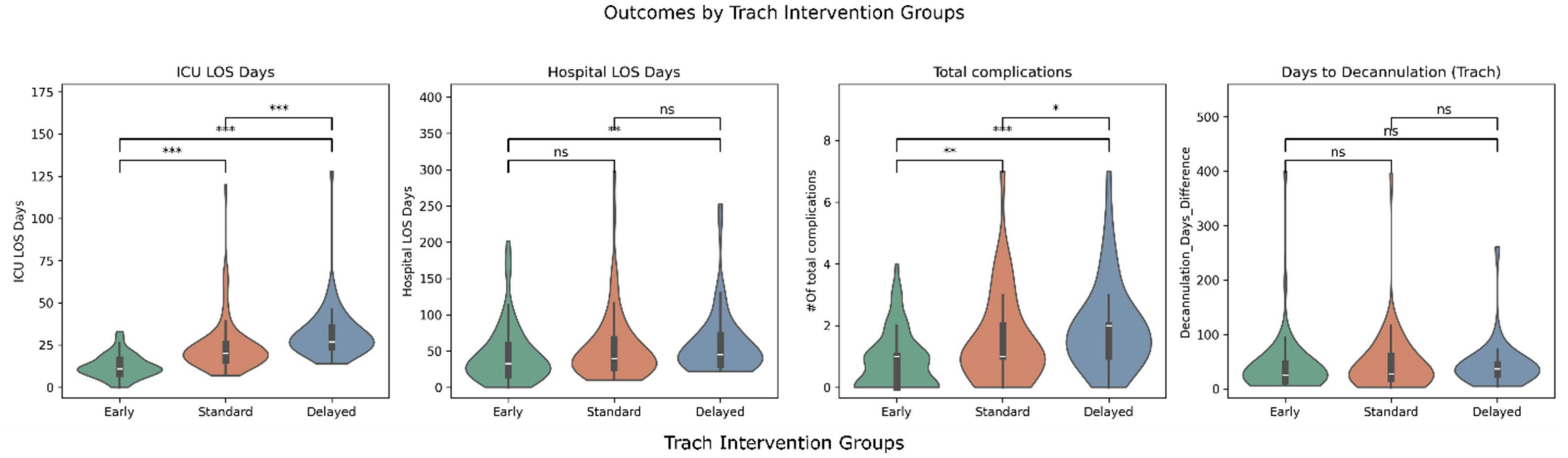
Outcomes by trach intervention groups (early, standard, delayed). *** = *p* < 0.001, ** = *p* < 0.01, *p* < 0.05, ns, not significant.

Figures 1, 2 illustrate the distribution of ICU LOS, hospital LOS, total complications, and time to oral feeding/decannulation across the intervening timing groups for both PEG and trach patients, respectively. Early intervention groups consistently demonstrated shorter LOS and total complications compared to the standard and delayed groups. Of note, the distribution of ICU LOS was tight in the early intervention group for both PEG and trach patients and became broader with delayed placement.

The days before oral feeding or decannulation did not show clear differences across the groups. Linear regression models confirmed these findings but found that GCS at admission was significant in predicting days to oral feeding (*β* = 4.63, *p* = 0.02). Similarly, ISS was a predictor of quicker decannulation (*β* = 0.65, *p* = 0.029).

A logistic regression analysis suggested that delayed PEG placement was associated with a significantly lower risk of mortality compared to earlier placement (OR = 0.33, *p* = 0.033); excluding patients with the top 10% CCI within the cohort yielded similar findings (OR = 0.35, *p* = 0.05). However, there was no significant relationship found between PEG timing and readmission rates. In the trach group, intervention timing was not linked with mortality (*p* > 0.1 for all groups) or readmission rates. However, older age was linked to higher mortality (OR: 1.04, *p* = 0.028), and higher NLR values were associated with an increase in readmission risk (OR: 1.07, *p* = 0.025). Delayed trach placement had a significantly increased risk of developing VAP (OR = 1.05, *p* = 0.016). Increased ISS was also associated with slightly higher VAP risk (OR: 1.03, *p* = 0.046), while other factors, such as age and GCS, were not significant predictors.

The Cox proportional hazards model for PEG and trach timing did not find significant associations with the time to readmission (*p* > 0.05 for all intervention groups), although age emerged as a significant predictor of earlier readmission in trach patients: older patients were readmitted quicker (HR: 1.03, *p* = 0.036). Notably, delayed PEG placement was associated with a lower risk of earlier mortality, although with borderline significance (HR: 0.93, CI: 0.87–1.00, *p* = 0.05).

The OLS regression implied that the timing of PEG and trach interventions demonstrated mixed effects on neurological recovery, as measured by the change in GCS from admission to discharge (‘Delta_GCS’). Each additional day of delayed PEG placement was significantly associated with a slight yet significant improvement in neurological function (*β* = 0.042, *p* = 0.039). Meanwhile, early trach placement had a slight negative association with GCS improvement (*β* = −0.08, *p* = 0.040), suggesting that early trach placement may not enable as much neurological recovery compared to delayed placement.

### Temporal difference learning

The reward function *R* had a specific reward/penalty scheme tabulated in Table 4.

**Table 4:** Overview of the reward function metrics for temporal difference and Q-learning.

Metric	Reward function *R*
$R_{\mathrm{ICU}\;\mathrm{LOS}}$	−ICULOSDays
$R_{\text{Hospital}\;\mathrm{LOS}}$	−HospitalLOSDays
$R_{\text{Admission}}$	$\left\{ \begin{array}{c} -10,\text{if Readmission}=\mathrm{Yes}\\ +5,\text{if Readmission}=\mathrm{No} \end{array}\right.$
$R_{\text{Mortality}}$	$\left\{ \begin{array}{c} -10,\text{if Mortality}=\mathrm{Yes}\\ +5,\text{if Mortality}=\mathrm{No} \end{array}\right.$
$R_{\text{Oral Intake}}$	$\left\{ \begin{array}{c} -0.5\times \text{Days to Oral Intake},\text{if delay}\geq 1\;\mathrm{day}\\ +3,\text{if}\;\mathrm{no}\;\text{delay}\; \end{array}\right.$
$R_{\mathrm{VAP}}$	$\left\{ \begin{array}{c} -5,\text{if}\;\mathrm{VAP}=\mathrm{Yes}\\ +3,\text{if}\;\mathrm{VAP}=\mathrm{No} \end{array}\right.$
$R_{\text{Decannulation}}$	$\left\{ \begin{array}{c} -0.5\times \text{Days to Decannulation},\text{if delay}\geq 1\;\mathrm{day}\\ +3,\text{if}\;\mathrm{no}\;\text{delay}\; \end{array}\right.$

For PEG patients, earlier intervention was associated with better overall outcomes, with a value function of −295.49; standard and delayed interventions showed progressively worse outcomes (more negative), with value functions of −505.44 and −536.36. Similarly, early intervention in trach patients had a higher value function (less negative) of −356.41, while standard and delayed had a less pronounced but worse value of −456.62 and −442.58, respectively. Uniform scaling of all reward magnitudes by ±50% changed absolute value magnitudes but did not alter the timing ranking for either PEG or trach (Supplementary Figure S1A). In one-at-a-time sensitivity tests (Supplementary Figure S1B), the oral-intake delay coefficient (for PEG) produced the largest change in value-function separation between the top two timings (Δgap ≈ 28.7), followed by the survival reward (Δgap ≈ 8.1) and the no-readmission reward (Δgap ≈ 5.7); other weights had minimal impact. For trach, the decannulation-delay coefficient had the largest effect (Δgap ≈ 43.9), followed by the readmission penalty (Δgap ≈ 11.7) and the no-VAP reward (Δgap ≈ 7.6). These sensitivity analyses suggest that learned recommendations are somewhat robust to reasonable changes in the clinical value weights.

**Figure 3:**
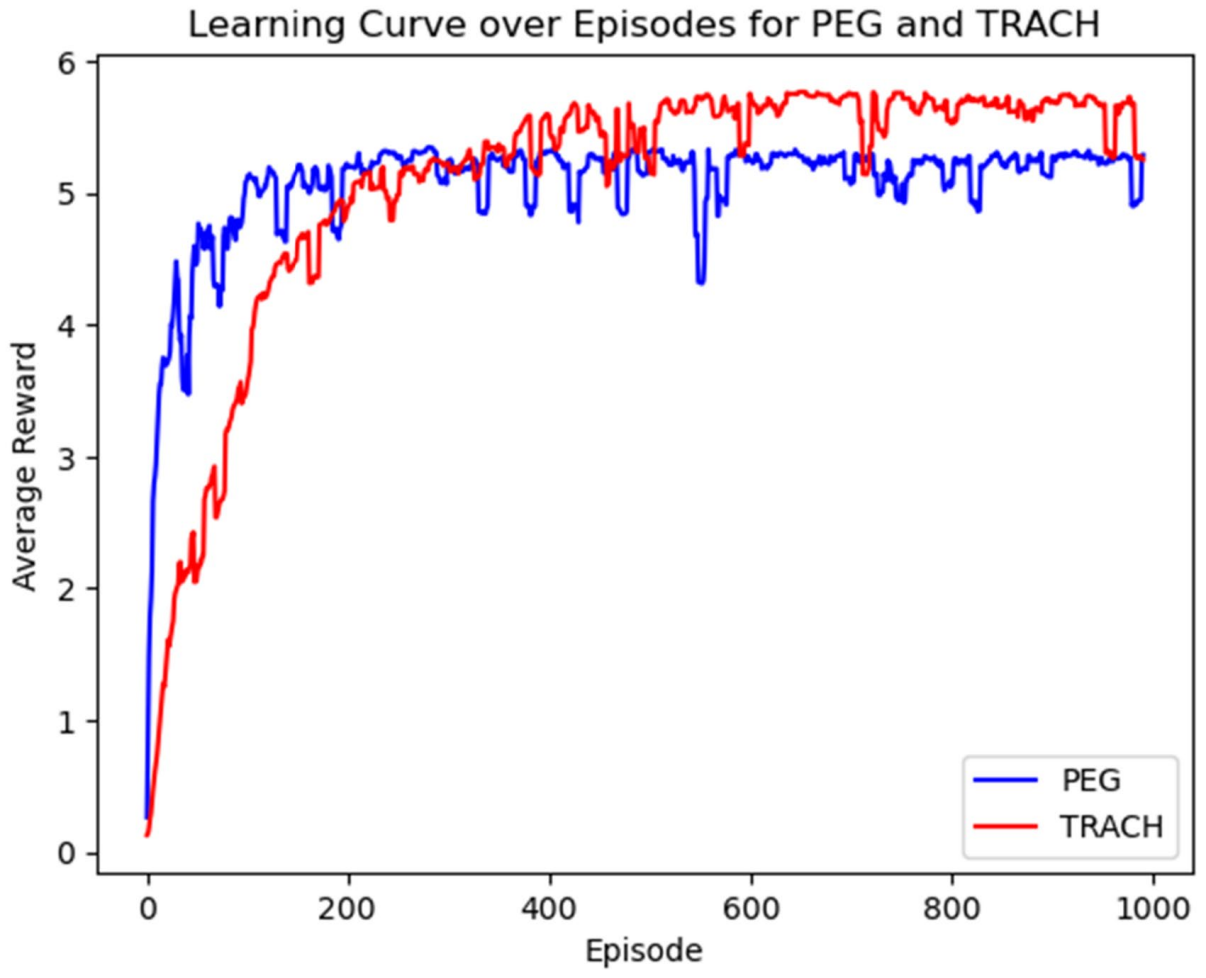
Average reward over 1,000 episodes for PEG and trach interventions by Q-learner.

The average reward per learning episode for both PEG and trach patients is shown in Figure 3, for over 1,000 episodes. The average reward for both groups improved as the model learned with each episode, suggesting the model’s increasing ability to identify optimal intervention timings. The model was especially able to quickly identify patterns for PEG timing and had a slower convergence for trach patients, suggesting that outcomes may be influenced by more factors or are harder to predict earlier in the learning process.

**Figure 4:**
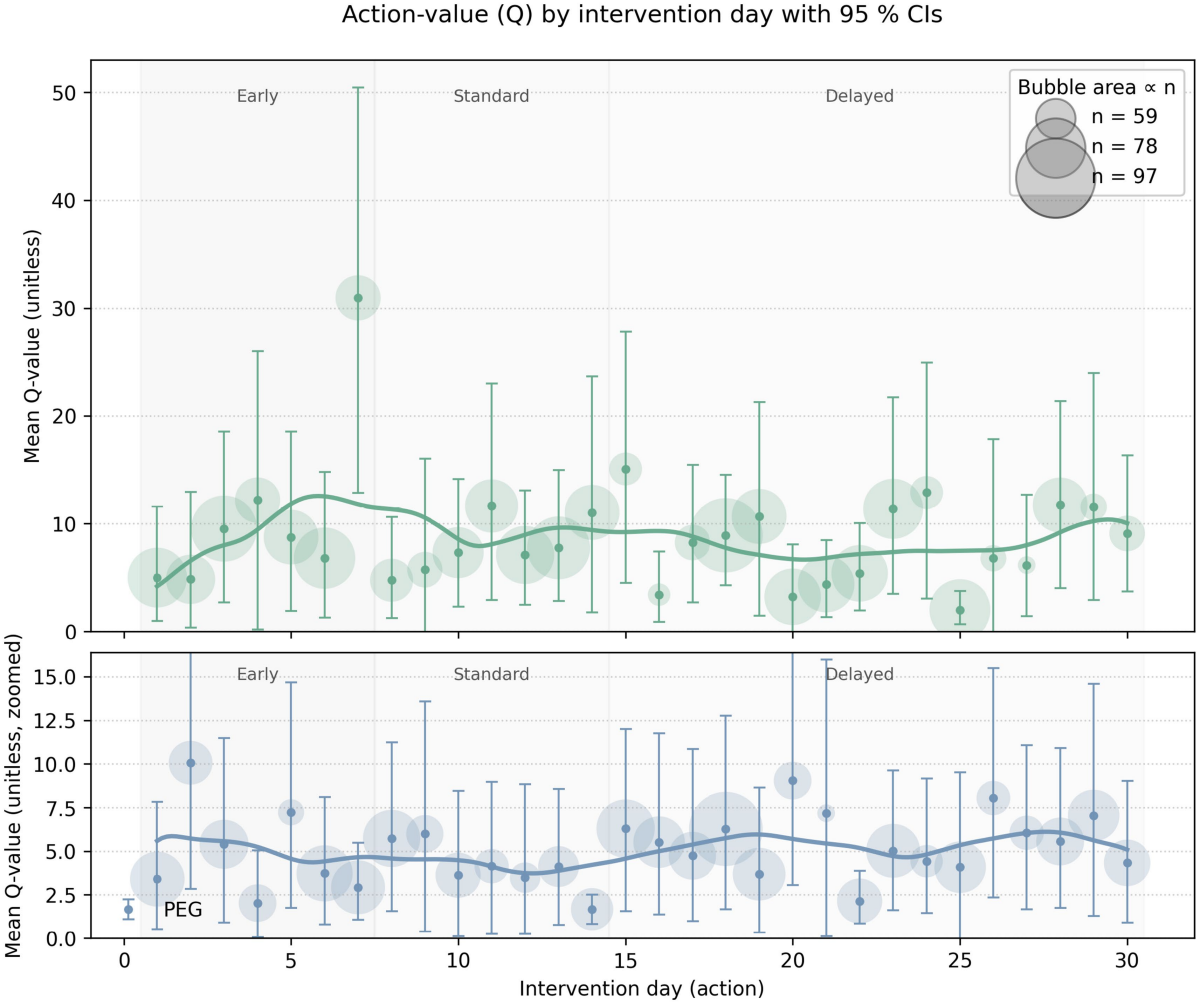
Visual representation of the Q-values for different intervention days for both PEG (blue) and trach (green) interventions. Bubble size corresponds to the sample; bars refer to the confidence interval/level of those Q-values; the y-axis represents the mean Q-value or the expected cumulative reward for intervening on a particular day.

A visualization of the Q-values (expected rewards) for different intervention days (0–30 days) for both PEG and trach is in Figure 4. The tracheostomy curve showed higher Q-values in the early period (days 3-7), while PEG exhibited a flatter, more nuanced trend across time. There were inconsistent confidence levels in the standard range of intervention timing for both PEG and trach. There were high Q-values in trach patients on days 20–30, indicating that delaying trach procedures in some cases may yield better cumulative rewards. On the other hand, PEG interventions had lower Q-values overall in the late period.

The Q-value confidence interval ranged from 5.90 to 8.23 for PEG patients and from 8.58 to 11.76 for trach patients. High-confidence policies recommended early PEG intervention for those with higher admission GCS scores and fewer comorbidities and often had a higher cumulative reward, with Q-values often exceeding 100. For trach patients, high-confidence policies similarly recommended early placement, particularly for patients with moderate ISS and younger age. Low-confidence policies were generally used for patients with a complex presentation, such as those with high comorbidity scores, lower GCS at admission, higher NLR values, or older age. Q-values for these patients were often close to 0, suggesting that the decision between earlier or late intervention may have been less impactful or more ambiguous.

**Figure 5:**
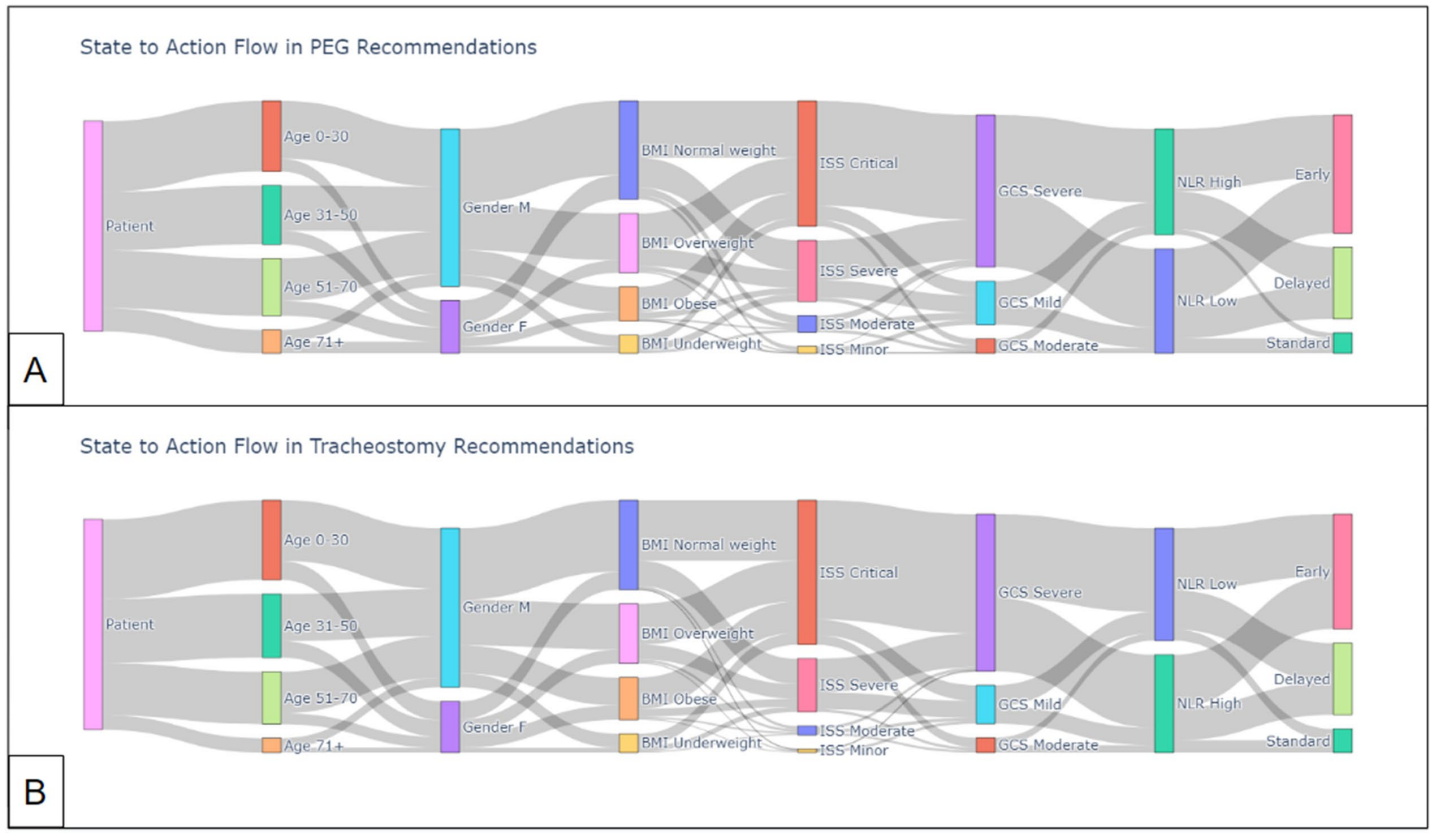
State-to-action flow by Q-learner for **(A)** PEG recommendations and **(B)** trach recommendations.

Figure 5 visualizes the flow from state (patient characteristics) to action (intervention timing) by the Q-learner for PEG and trach patients.

## Discussion

A primary goal of this study was to assess whether early intervention leads to better clinical outcomes. Early PEG and trach placement consistently resulted in shorter ICU and hospital stays, aligning with prior findings that suggest that early intervention in TBI management is often beneficial in terms of resource utilization and patient recovery (6, 7). As intervention timing shifted from early to standard or delayed, the distribution of the ICU LOS broadened, reflecting that delayed interventions could either result from worsening clinical conditions or contribute to more complications. Furthermore, early intervention groups for both PEG and trach consistently had lower total complication rates, especially VAP in trach patients. In agreement with de Franca et al.’s meta-analysis, delayed trach placement was found to increase the risk of developing VAP, which is not unexpected, as prolonged mechanical ventilation would increase the likelihood of lung infection (6).

Interestingly, delayed PEG placement was associated with a 67% reduction in the odds of mortality compared to early placement despite increased total complication rates (OR = 0.33, *p* = 0.033); the relationship marginally persisted after excluding the top 10% of patients with the highest comorbidity index. The finding is moderately robust, although residual confounding from unmeasured severity, treatment-limitation decisions, and timing biases cannot be excluded. For example, the low ISS within the same group may contribute; however, the trend was consistent with patterns learned by the reinforcement learning (RL) model, which incorporated ISS within its state space and similarly suggested delayed PEG as beneficial in specific patients. This finding contrasts with the findings by Figueiredo et al. and is rather much more consistent with concerns raised by Chaudhry et al. (20, 24). Their population-based study, which had similar time categorizations as our study, found that late PEG placement (after 2 weeks) was indeed linked with a higher incidence of complications, such as sepsis, urinary tract infections, or acute respiratory distress syndrome (ARDS), but had a decreased mortality risk in high-risk groups. On the other hand, early PEG placement, despite being performed on patients with fewer comorbidities, was paradoxically associated with higher in-hospital mortality. Standard PEG timing (7–14 days) was the most appropriate to mitigate mortality and complications in low- and moderate-risk groups.

These findings could be explained by the fact that late PEG may often be performed in patients with higher comorbidity burdens: clinicians are likely to delay the procedure until patients are clinically stable to mitigate the mortality risk. In some cases, early intervention may still be premature and result in unforeseen complications in patients who appear to be initially stable. For example, each additional day of delayed PEG placement was linked with a slight yet significant improvement in neurological function, reinforcing that delaying PEG in some cases may allow patients to stabilize. This finding was also observed in our findings with trach placement, where early intervention was linked to slower neurological recovery, as seen with the slight negative association with GCS improvement.

The trends in our study, when understood in the context of prior literature, collectively imply the need to personalize the timing of both PEG and trach procedures in TBI patients. The general benefits of early intervention may not be visualized across all patients, and the decision to perform early, standard, or delayed interventions should still consider individual patient factors that could significantly influence their trajectories, as seen in our covariate analyses. For example, patients who were older or had an increased NLR at admission were more vulnerable to readmission or mortality after intervention. Notably, NLR has gained attention as a biomarker to provide insight into the recovery of TBI patients (25). The NLR describes the balance between neutrophil and lymphocyte counts and has been considered an indicator of systemic inflammation. Elevated NLR is supposedly linked to a heightened inflammatory response, which can increase secondary brain injury in TBI patients. For example, TBI patients with higher NLR values at admission were more likely to experience complications that could require prolonged mechanical ventilation or delayed recovery (25).

The complexity of determining the optimal timing of intervention positions RL to offer a unique approach that can integrate patient-specific factors and support the clinician’s decisions. Our study used TD learning and a Q-learner to estimate the net cumulative benefit of these interventions based on the historical patient data and subsequently learn optimal intervention strategies. The value function derived from TD learning agreed with the general trend of how earlier interventions resulted in greater rewards. In PEG patients, younger individuals and those with fewer comorbidities, as quantified by the Charlson Comorbidity Index, had the best overall value with early intervention. For trach patients, although the value function pointed to earlier intervention to maximize outcomes, there was no observable pattern in relation to specific patient characteristics, suggesting a much more nuanced pattern.

The Q-learner was able to successfully explore the data and develop decision-making capability, as demonstrated by the reward over time curve (Figure 3). The model was able to quickly identify patterns for PEG timing and had a slower convergence for trach patients, suggesting that outcomes may be influenced by more factors or are harder to predict earlier in the learning process. The Q-learner reflected some established trends while offering novel insights, such as how early interventions were generally preferred, especially among younger patients. Meanwhile, older patients, especially those over 71, received a mix of early, standard, and delayed intervention recommendations, mirroring the cautious approach to older populations, as they may be more vulnerable to risks. Patients with a BMI above 25 or presenting with a severe/critical ISS were more likely to be directed to a delayed recommendation. Those with moderate ISS were more likely to receive earlier intervention recommendations. The Q-learner also implied that patients presenting with severe GCS tended to receive delayed intervention recommendations, while those with mild to moderate GCS scores exhibited a more varied pattern. Patients with a higher NLR were often recommended a later date for intervention compared to those with a lower NLR.

While the application of Q-learning in our study is the first of its kind in developing clinical decision support systems for TBI management, there are some scalability issues to consider. The model was trained on historical data from a single institution, which may limit its generalizability. Practices at a single academic institution, such as ICU protocols and surgeon experience, may limit the external validity of our model. Q-learning’s constraint as an offline learner restricts the model from freely exploring a decision space in the real-world setting; it is unethical to test interventions on actual patients to learn the best action (23). At the same time, this makes the model vulnerable to extrapolation bias as it learns from static, retrospective datasets; ethical deployment would require human-in-the-loop validation. Although exploration enables the model to experiment with different actions, as seen in Figure 3 with the occasional dips in reward, this aspect could nonetheless result in extrapolation error. Striking the balance in the exploration-exploitation tradeoff as an epsilon-greedy policy can be challenging, which is why it is essential to continue the model training on diverse, up-to-date, and prospective datasets to improve the model and better assess its performance before a full-scale deployment. Adding more features to the state space or updating the reward function for long-term outcomes may also expand the depth of the study. Future research can evaluate novel off-policy RL approaches or simulations to limit extrapolation error in optimizing TBI management (26).

## Conclusion

In conclusion, the association between the timing of intervention and improved outcomes in TBI patients is a question of precision medicine. Early PEG and trach interventions in TBI patients generally optimize resource allocation and some outcomes, including shorter ICU/hospital stays and fewer complications such as VAP. However, the link between intervention timing and mortality or neurological recovery is likely more nuanced, with delayed PEG placement linked to improved survival in select cases. Applying RL techniques may show promise in optimizing intervention timing based on patient characteristics, and further validation and refinement are needed in this novel strategy. Future studies should build upon the work in this study with larger datasets and stronger RL algorithms to improve decision-making in TBI management.

## Data Availability

The raw data supporting the conclusions of this article will be made available by the authors upon request, without undue reservation.
