# Supplementary figures and images for: Can one-step reinforcement learning guide optimal timing for PEG and tracheostomy in severe TBI? Insights from a 2016–2023 retrospective cohort study at a single academic institution
Source: Front Neurol. 2025 Nov 13;16:1700064. doi: 10.3389/fneur.2025.1700064 (PMC12657412; doi:10.3389/fneur.2025.1700064)

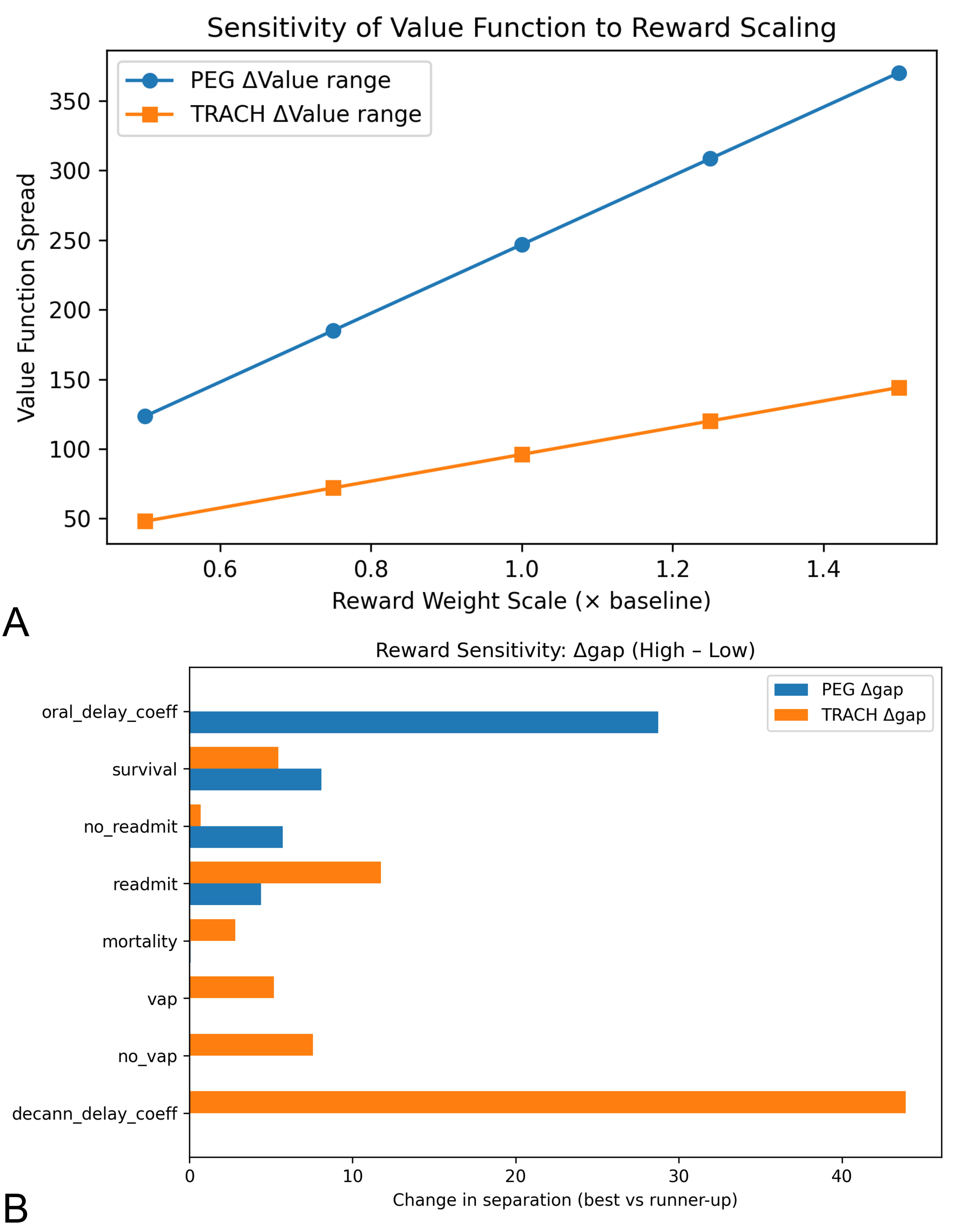

Supplement: Supplementary file 1 [file Image_1.PNG]
